# Terahertz Radiation from Combined Metallic Slit Arrays

**DOI:** 10.1038/s41598-019-43072-2

**Published:** 2019-05-02

**Authors:** Dazhi Li, Makoto Nakajima, Masahiko Tani, Jinfeng Yang, Hideaki Kitahara, Masaki Hashida, Makoto Asakawa, Wenxin Liu, Yanyu Wei, Ziqiang Yang

**Affiliations:** 1 Institute for Laser Technology, Osaka, 5650871 Japan; 2 Institute of Laser Engineering, Osaka University, Osaka, 5650871 Japan; 3 Research Center for Development of Far-Infrared Region, University of Fukui, Fukui, 9108507 Japan; 4 The Institute of Scientific and Industrial Research, Osaka University, Osaka, 5670047 Japan; 5 Advanced Research Center for Beam Science, ICR, Kyoto University, Kyoto, 6110011 Japan; 6 Faculty of Engineering Science, Kansai University, Osaka, 5648680 Japan; 7 Key Laboratory of High Power Microwave Sources and Technologies, Institute of Electronics, Chinese Academy of Sciences, Beijing, 100190 China; 8 School of Electronics Science and Engineering, University of Electronic Science and Technology of China, Chengdu, 610054 China

**Keywords:** Terahertz optics, Electronics, photonics and device physics

## Abstract

We report an approach to efficiently generate terahertz radiation from a combined periodic structure. The proposed configuration is composed of two metallic slit arrays deliberately designed with different periodic length, slit width and depth. We found that the combination of the two slit arrays could provide special electromagnetic modes, which exhibit nonradiative property above the surface of one slit array and radiative property inside the other one. An electron beam holding proper energy could resonate with those modes to generate strong and directional electromagnetic radiations in the terahertz regime, indicating that the approach has the potential in developing high-performance terahertz radiation sources.

## Introduction

There is continued interest in developing terahertz radiation sources^1–14^ to meet various requirements of increasing applications^15–18^ in scientific and industrial fields. In our previous work^19^, a composite structure of a metallic slit array attached with a dielectric substrate was proposed to produce enhanced terahertz wave based on a special radiation mechanism. The composite structure has the ability to generate radiative eigen mode, which is possible to interact with the electrons traveling over the surface of the slit array^19^. The interaction induces a resonant radiation mechanism, which can be expected to develop high-efficiency terahertz radiation sources. However, there are limitations in choosing materials for the dielectric substrate in extending such a configuration to high power terahertz device, due to the thermal issues^20^ and dielectric breakdown^21^. Besides, the possibility of the dielectric properties destroyed by the scattered electrons bombarding the substrate increases with the increase of the electron beam current in pursuing powerful radiation sources. Challenges, therefore, still exist to find an alternative scheme to overcome those barriers.

The use of an all-metal structure is a straightforward solution to the aforementioned problems, as the metals are more robust than the dielectric materials against those limitations. With the development of electromagnetic metamaterials^22–25^ and nanophotonics^26–28^ in recent years, it becomes possible to construct metallic devices with desired optical properties. Along with this line, we propose a configuration comprised of two metallic slit arrays to realize resonant radiation in the terahertz regime. The two slit arrays are designed with different dimensions in order to realize different optical properties. And the combination of the two assemblies could provide radiative electromagnetic modes that can resonate with an incident electron beam to generate enhanced terahertz radiation. Moreover, the configuration exhibits a special refractive property, and we use it ingeniously to design a high-performance output system.

## Results

### Configuration and electromagnetic modes

**Figure 1:**
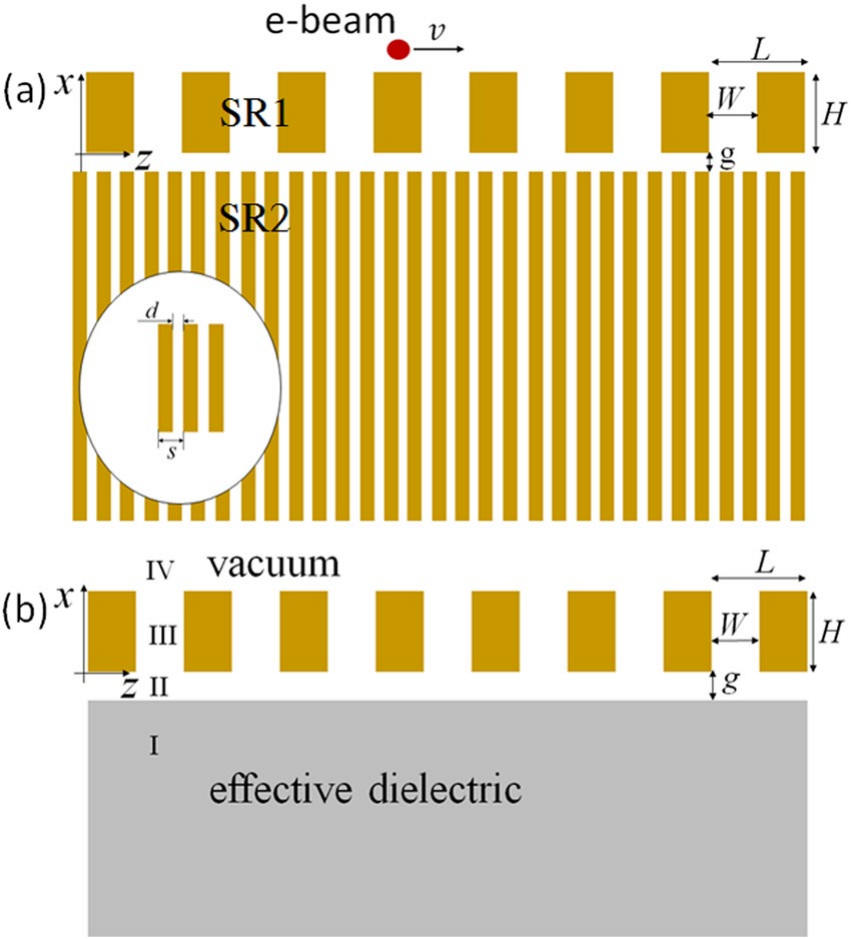
(**a**) Schematic diagram of a configuration with combined slit arrays, and (**b**) its corresponding effective model for theoretical analysis.

The configuration is composed of two slit arrays, labeled as SR1 and SR2, respectively, as shown in Fig. 1(a). Each of them is a one-dimensional array and considered to be made of a perfect conductor. SR1 serves as a slow-wave structure that has been widely used in Smith-Purcell free-electron lasers^3–6^, while SR2 is designed to be a sub-wavelength structure, with the periodic length being much smaller than the operating wavelength^29^. For assembly, a narrow gap is set between SR1 and SR2 to benefit the field coupling between adjacent slits. We developed a two-dimensional theory to describe the electromagnetic properties of the system. The periodicity of these slit arrays is chosen in the *z* direction, whereas the slits are parallel to the *y* axis. The dimensions for SR1 and SR2 are designed respectively as: L = 0.2 mm, W = 0.1 mm, H = 0.4 mm, s = 50 μm, and d = 12.5 μm, so that the radiation waves occur in the terahertz regime. The gap between SR1 and SR2 is assigned to be g = 10 μm. The sub-wavelength slit array is usually described by two different effective dielectric theories. One maps the structure into an isotropic dielectric medium with an effective refractive index, *n*_*i*_ = s/d^30^. And the other one gives an anisotropic description^31^, and it characterizes the permittivity by *ε*_*z*_ = s/d, and *ε*_*x*_ = ∞. The two theories have been examined through particle-in-cell simulation method^32^, and it is addressed that the latter agrees much better to the simulation results. An effective dielectric model for SR2 is used to simplify our theoretical analysis, as shown in Fig. 1(b). We incline to adopt the effective permittivity *ε*_*a*_ = s/d, and this results in ${n}_{a}=\sqrt{{\varepsilon }_{a}}=2$ with the parameters mentioned earlier. Actually, we have to modify this value to make our theoretical analysis more precise. As has been addressed in ref.^32^, the radiation angle of a Cherenkov-like radiation induced by an electron traveling along the surface of a sub-wavelength structure with velocity *v* should follow $\tan \,{\theta }_{c}=v/c$, where *θ*_*c*_ is the radiation angle to the electrons’ traveling direction and *c* is the light velocity in vacuum, while the Cherenkov radiation angle in a normal dielectric medium is given as $\cos \,{\theta }_{c}=c/nv$, where *n* is the refractive index of the dielectric medium. In order to correspond to the radiation angle in sub-wavelength slit array, the refractive index of an effective dielectric medium is derived as ${n}_{ef}=c/v\,{\cos }(\arctan (v/c))$, which relates to the electron’s velocity. A 100-keV electron bunch is adopted for all calculations in this paper. With using this value, we can work out the effective refractive index as *n*_*ef*_ = 2.08, which is a little bit larger than *n*_*a*_. Then, the effective permittivity is obtained as ${\varepsilon }_{ef}={n}_{{ef}^{2}}=4.33$. We use *n*_*ef*_ and *ε*_*ef*_ in the following analysis.

We focus on the dispersion relation of this configuration, and our attention is confined to transverse magnetic (TM) waves, for which the magnetic field in the *z*-direction vanishes. The TM wave is usually used in the electromagnetic wave sources driven by an electron beam, such as backward-wave oscillators, traveling-wave tubes and Smith-Purcell free-electron lasers, because it holds the longitudinal electric field that can interact with the electron beam^3–6^. Assuming the time dependence *e*^−*jωt*^ for all field components, the *y*-directed magnetic field and the *z*-directed electric field in the dielectric region (region I in Fig. 1(b)) can be expressed as

1 $${H}_{I,y}(x,z)=\sum _{p=-\infty }^{\infty }{A}_{I,p}{e}^{-j{\alpha ^{\prime} }_{p}x}{e}^{j{k}_{p}z},$$

2 $${E}_{I,z}(x,z)=\frac{1}{\omega {\varepsilon }_{ef}{\varepsilon }_{0}}\sum _{p=-\infty }^{\infty }{A}_{I,p}{\alpha ^{\prime} }_{p}{e}^{-j{\alpha ^{\prime} }_{p}x}{e}^{j{k}_{p}z},$$

where ${\alpha ^{\prime} }_{p}=\sqrt{{\varepsilon }_{ef}{\omega }^{2}/{c}^{2}-{k}_{p}^{2}}$, *kp* = *k*_*z*_ + 2*πp*/*L*, and *A*_*I*,*p*_ is scalar coefficient to be determined. In the vacuum gap (region II) between the slit array and the dielectric, the fields are written as

3 $${H}_{II,y}(x,z)=\sum _{p=-\infty }^{\infty }({A}_{I{I}^{+},p}{e}^{-j{\alpha }_{p}x}+{A}_{I{I}^{-},p}{e}^{j{\alpha }_{p}x}){e}^{j{k}_{p}z},$$

4 $${E}_{II,z}(x,z)=\frac{1}{\omega {\varepsilon }_{0}}\sum _{p=-\infty }^{\infty }({A}_{I{I}^{+},p}{e}^{-j{\alpha }_{p}x}-{A}_{I{I}^{-},p}{e}^{j{\alpha }_{p}x}){\alpha }_{p}{e}^{j{k}_{p}z},$$

where ${\alpha }_{p}=\sqrt{{\omega }^{2}/{c}^{2}-{k}_{p}^{2}}$, ${A}_{I{I}^{+},p}$ and ${A}_{I{I}^{-},p}$ are scalar coefficients to be determined. We only consider the fundamental mode in the slit (region III), and the reasonability of this consideration has been specified in ref.^3^. So, the fields in the slit are written as

5 $${H}_{III,y}(x,z)={A}_{II{I}^{+}}cos(\frac{\omega }{c}x)+{A}_{II{I}^{-}}sin(\frac{\omega }{c}x),$$

6 $${E}_{III,z}(x,z)=\frac{j}{c{\varepsilon }_{0}}(-{A}_{II{I}^{+}}sin(\frac{\omega }{c}x)+{A}_{II{I}^{-}}cos(\frac{\omega }{c}x)),$$

where ${A}_{II{I}^{+}}$ and ${A}_{II{I}^{-}}$ are scalar coefficients to be determined. In the vacuum region (region IV) above the slit array, the fields can be expressed as

7 $${H}_{IV,y}(x,z)=\sum _{p=-\infty }^{\infty }{A}_{IV,p}{e}^{j{\alpha }_{p}x}{e}^{j{k}_{p}z},$$

8 $${E}_{IV,z}(x,z)=-\,\frac{1}{\omega {\varepsilon }_{0}}\sum _{p=-\infty }^{\infty }{A}_{IV,p}{\alpha }_{p}{e}^{j{\alpha }_{p}x}{e}^{j{k}_{p}z},$$

where ${A}_{IV,p}$ is scalar coefficient to be determined. By making use of the boundary conditions, the dispersion relation between angle frequency *ω* and wavenumber *k*_*z*_ can be directly derived as

9 $$\sum _{p=-\infty }^{\infty }\tfrac{j\omega ({\sin }(\tfrac{\omega }{c}H)\xi -\,{\cos }(\tfrac{\omega }{c}H)){\zeta }^{2}}{c{\alpha }_{p}L}=W({\cos }(\frac{\omega }{c}H)\xi +\,{\sin }(\frac{\omega }{c}H)),$$

where

$$\begin{array}{rcl}\xi  & = & \frac{1}{W}\sum _{p=-\infty }^{\infty }\frac{j\omega {\zeta }^{2}(1+{\rm{\Gamma }})}{c{\alpha }_{IV,p}L(1-{\rm{\Gamma }})}\\ {\rm{\Gamma }} & = & \frac{{e}^{j2d{\alpha }_{p}}({\varepsilon }_{ef}{\alpha }_{p}-{\alpha ^{\prime} }_{p})}{{\varepsilon }_{ef}{\alpha }_{p}+{\alpha ^{\prime} }_{p}}\\ \zeta  & = & \frac{2}{{k}_{p}}\,\sin (\frac{{k}_{p}W}{2})\end{array}$$

**Figure 2:**
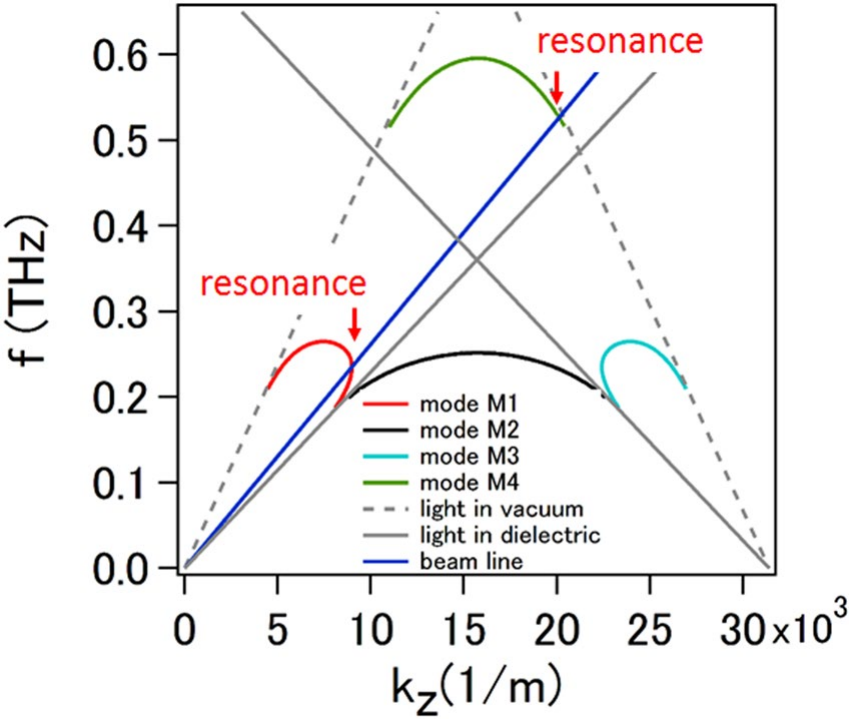
Dispersion relation of the effective model of combined slit arrays (resonance occurs at the intersection of the beam line with the curve of mode M1 and M4, respectively).

With the parameters given earlier, we numerically solved equation (9) and obtained four modes denoted as M1, M2, M3 and M4 in Fig. 2. The light line in vacuum $f=c{k}_{z}/2\pi$, and in effective dielectric $f={k}_{z}c/2\pi {n}_{ef}$ are also plotted in Fig. 2 for comparison. It is shown that mode M2 is a classic evanescent mode located in the region below the light line of dielectric, which means it cannot radiate both in vacuum and dielectric; mode M1, M3 and M4 exist above the light line of dielectric but below that of vacuum, which means that they can radiate in the dielectric side and operate as surface waves in the vacuum region. A 100-keV electron beam can interact with the mode M1 and M4, and we also plotted the electron beam line $f={k}_{z}v/2\pi$. From Fig. 2 we know that the electron beam can excite radiative waves in the dielectric region, and these waves emit at the angle determined by $cos{\theta }_{c}=c/{n}_{ef}v$, which exhibits the property of Cherenkov radiation. At the intersection of the beam line with the curve of the mode M1 and M4, resonance happens and hence the enhanced radiation at the resonant frequency can be predicted. The resonant frequency is calculated to be 0.23 THz for the mode M1 and 0.52 THz for the mode M4, respectively. The radiation angle for both of the radiations is 28.73 degrees with respect to the travelling direction of the electron beam, which can be obtained through numerically solving the dispersion equations with using the given parameters. Note that the radiation is generated and propagates only in the lower slit array, and originally it doesn’t appear in the vacuum region above the upper slit array.

**Figure 3:**
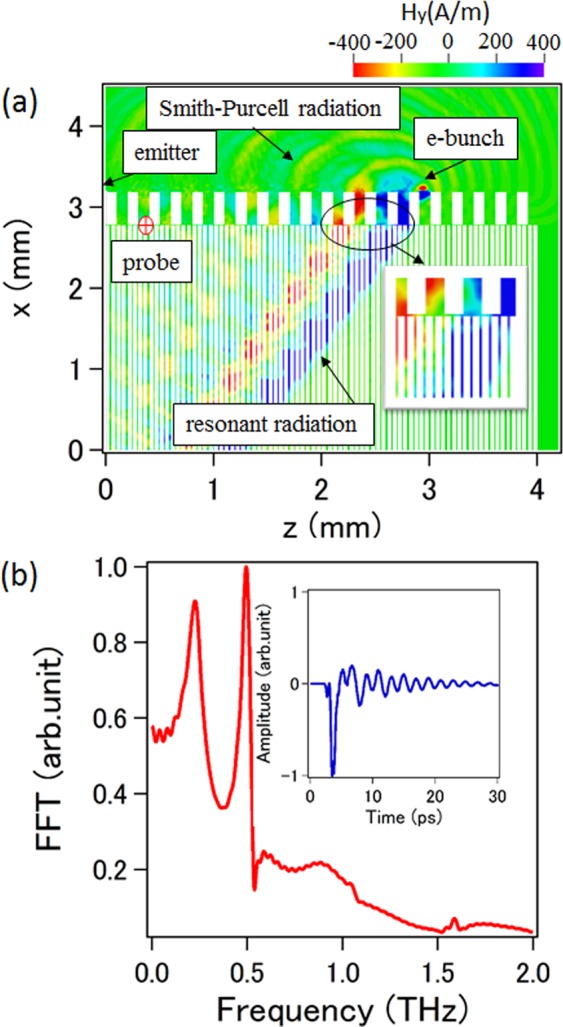
(**a**) Simulation results for the contour map of the *y-*directed magnetic field. (**b**) The radiation spectrum together with the corresponding time-domain waveform observed at the probe point.

We performed computer simulations to demonstrate theoretical predictions with the help of a particle-in-cell code^33^. Twenty and eighty periods are considered for SR1 and SR2, respectively, for the simulation model, and the other parameters are same as those mentioned earlier. In order to understand the radiation characteristics in such a configuration, we make a single electron bunch with a length of 0.2 ps and a charge of 0.2 pC pass over the surface of SR1. The distance between the bunch and the surface of SR1 is set to be 10 μm, so that the electromagnetic waves can be excited effectively. Two-dimensional simulations were performed and the results are given in Fig. 3. From the contour map of the *y*-directed magnetic field shown in Fig. 3(a), we can clearly see that a strong wave generates from the region between SR1 and SR2, and it emits in the region of SR2 at a specific direction corresponding to the angle predicted earlier. It should be noted that the wave can only propagate vertically in the slit with light velocity, and the radiation wave seen in SR2 is actually formed by those waves from separate slits. The temporal behavior of the *y-*directed magnetic field along with the corresponding finite Fourier transformed (FFT) spectrum observed at the point noted as probe is given in Fig. 3(b). Two peaks indicating the resonances appear at frequency 0.22 THz and 0.49 THz, respectively, which agree well with the theoretical predictions. From Fig. 3(a) we know that the resonant radiation does not radiate above the surface of SR1, just acting as a surface wave propagating along SR1. Besides, the electron beam also induces normal Smith-Purcell radiation as seen in Fig. 3(a), which is much weaker than the resonant radiation, and will not be discussed in this paper.

### Output scheme

**Figure 4:**
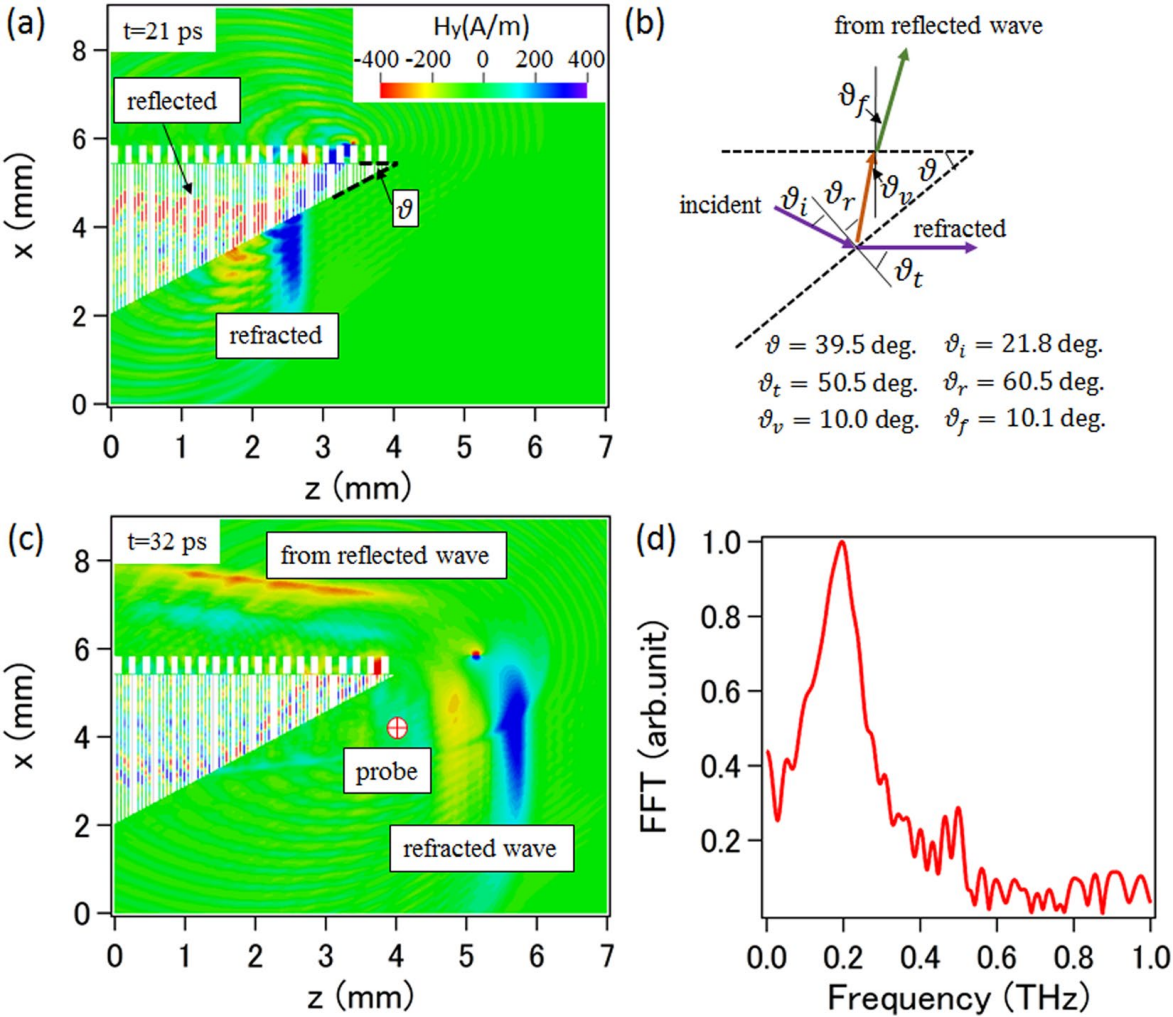
(**a**) Simulation results for the contour map of the *y-*directed magnetic field at t = 21 ps. (**b**) Schematic diagram of the radiation scheme. (**c)** Simulation results for the contour map of the *y-*directed magnetic field at t = 32 ps. (**d**) Radiation spectrum observed at the probe point.

**Figure 5:**
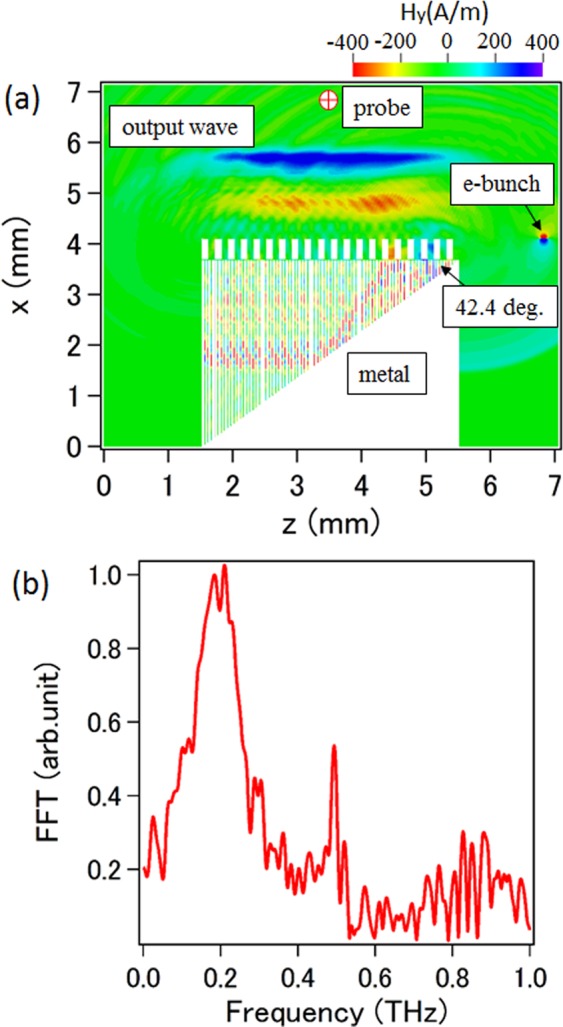
(**a**) Simulation results for the contour map of the *y-*directed magnetic field for reflection output system. (**b**) Radiation spectrum observed at the probe point.

We have to extract the radiation wave from SR2 so that they can be used. It is straightforward to consider an output scheme as shown in Fig. 4(a), where a slit array with wedge form is adopted. With using Snell’s law and the effective refractive index *n*_*ef*_ = 2.08, the wedge angle *ϑ* is designed to be 39.5 degrees, as shown in Fig. 4(a). Thus, the radiation wave propagating in SR2 is incident on the SR2-vacuum boundary at an incident angle *ϑ*_*i*_ = 21.8 degrees, and then the refracted angle *ϑ*_*i*_ = 50.5 degrees is obtained, and this leads to the refracted wave going in the z-direction, as shown in Fig. 4(b). Simulations are performed with the same parameters mentioned earlier, and the evolution of the distribution of the *y*-directed magnetic field with time is given. A snapshot at 21 ps from the beginning of the simulation is shown in Fig. 4(a), where the incident wave going through the boundary can be seen. Figure 4(c) shows a snapshot at 32 ps, where we see the refracted wave propagating in the vacuum region with the direction as designed. The frequency spectrum observed at the probe point is given in Fig. 4(d), showing that the radiation of 0.22 THz is predominant. Our best guess is that the period length adopted for the subwavelength structure SR2 is small enough for the wavelength of the low-frequency radiation and thus the subwavelength structure works well resulting in an effective output, while it is not enough for the high-frequency radiation. Though the refracted wave follows the Snell’s law, the reflected wave doesn’t follow the reflection law, as shown in Fig. 4(b), due to the fact that the propagation of each separate wave only occurs in the vertical direction inside each slit with the velocity of light. Considering the time difference for each separate wave reflected at the end of each slit and the geometrical structure, the reflection angle *ϑ*_*r*_ is calculated as 60.5 degrees, meaning that the reflected wave propagates in *ϑ*_*v*_ = 10.0 degrees to the vertical direction. In this case, the reflected wave can be considered to propagate in a velocity of ${v}_{v}=c\,\cos \,{\vartheta }_{v}$, which means it feels an effective refractive index of ${n}_{v}=1/cos{\vartheta }_{v}=1.02$. The reflected wave is incident on the SR2-gap boundary at an incident angle *ϑ*_*v*_ = 10.0 degrees, and then go through the boundary with a refractive angle *ϑ*_*f*_ = 10.1 degrees and form the output wave. According to the propagation property of the wave inside the slit array, we designed an output scheme based on the reflection effect as shown in Fig. 5. The slit array is considered to be a wedge form and the end of each slit is closed. The wedge angle is determined by $\vartheta ={\rm{arccot}}(2v/c)$, so that the reflected wave formed by separate waves in each slit going upward with light velocity, meaning that the reflected wave feels a refractive index 1. With the parameters mentioned earlier, the wedge angle is calculated to be 42.4 degrees. The contour plot of Hy is given in Fig. 5, where we see that the radiation wave goes vertically out of the configuration as expected. The frequency spectrum observed at the probe point is given in Fig. 5(b), and it is also shown that the radiation of 0.22 THz is predominant.

## Conclusion

The traditional electron-beam-driven devices, such as backward-wave oscillators, traveling-wave tubes and Smith-Purcell free-electron lasers, are usually considered to be high-power, continuous wave and compact terahertz radiation sources, and attempts are still being made to increase the average power, extend the frequency regime and improve the output performance. As is known, in such kind of devices the electron beam with medium energy is used to interact with the surface wave generated from a metallic periodic structure. The surface wave is a kind of nonradiative electromagnetic modes, which propagates along the surface of the structure, and it radiates only at the ends of the structure by the diffraction effect. In this paper, we considered a special electromagnetic mode generated by combined slit arrays, which are assembled by two slit arrays with different dimensions. This kind of mode exhibits the property of surface wave above the upper slit array, which can be exactly utilized to effectively realize sustaining interaction with the electron beam, and radiative property inside the lower slit array. The radiation mechanism is therefore different from that of traditional devices. Though the special mode can also be generated by a dielectric structure proposed in our previous work (ref.^19^), it is hard to extend the dielectric structure to high-power radiation source because of the thermal issues, dielectric breakdown and other problems. The proposed configuration of combined slit arrays is all-metal structure, and therefore can overcome those barriers to realize high power output. As addressed before, because the radiation occurs from the resonance of the special mode and the electron beam, single wavelength radiation can be generated and the wavelength could be continuously tuned by varying the electron-beam energy. From the dispersion relation shown in Fig. 2, we see that the resonances could occur on the downward sloping portion of the dispersion curve, meaning that the scheme can be developed to a coherent radiation source driven by an initial continuous electron beam^19^. From the contour plot of the radiation field obtained by simulations, we know that the radiation beam diverges slowly and it propagates in a certain direction. Therefore, it is easy to collect the radiation beam via classical optics, such as parabolic mirrors and lens. Consequently, based on the presented radiation system one can consider to develop tunable, single wavelength and directional terahertz emitters with using a single electron bunch, and to improve the performance of the traditional radiation devices or develop novel coherent radiation sources with using an initial continuous electron beam.

## Data Availability

All data generated or analyzed during this study are included in this article.
